# Classification of *Trypanosoma brucei* mammalian life cycle stages using Deep Learning Algorithms

**DOI:** 10.1371/journal.pntd.0013298

**Published:** 2025-08-14

**Authors:** Hamid Cheraghi, Lara López-Escobar, José Rino, Luisa M. Figueiredo, Bálint Szabó

**Affiliations:** 1 Department of Biological Physics, Eötvös Loránd University (ELTE), Budapest, Hungary; 2 CellSorter Scientific Company for Innovations, Budapest, Hungary; 3 Gulbenkian Institute for Molecular Medicine, Lisbon, Portugal; University of California Berkeley, UNITED STATES OF AMERICA

## Abstract

Accurate classification of *Trypanosoma brucei* bloodstream forms, slender and stumpy, is essential for understanding parasite biology and transmission dynamics. Traditional classification methods rely on flourescent transgenic parasites, as distinguishing these forms visually is highly challenging. To address this, we developed a semi-automated deep-learning pipeline that segments and classifies *T. brucei* bloodstream forms from unlabeled microscopic images. The pipeline consists of two key stages: (1) a segmentation step using the Cellpose algorithm, which detects and extracts individual parasites while filtering out artifacts, and (2) a classification step utilizing a deep learning model based on the Xception architecture. The classification model, optimized through transfer learning and fine-tuning, achieved a 97% accuracy, outperforming standard architectures such as InceptionV3, ResNet50, and VGG16. Our results demonstrate the effectiveness of deep learning in parasite stage classification, offering a scalable and efficient approach for high-throughput analysis. Beyond *T. brucei,* our framework can be adapted for other single-cell classification tasks based on unlabeled morphology, contributing to advancements in biomedical imaging and automated cell analysis.

## Author summary

*Trypanosoma brucei* is a parasite responsible for African sleeping sickness in humans and animals. Identifying the different stages of its life cycle is essential for understanding the disease and its progression, but this process often requires skilled experts and high-quality microscope images. In our study, we developed an automated method using artificial intelligence to classify the two main forms of the parasite—slender and stumpy—based on microscope images. Our approach combines a powerful segmentation tool called Cellpose with a deep learning model to analyze thousands of individual parasite images. Importantly, our method works well even with lower-resolution images, which are common in resource-limited settings. This makes the tool especially useful in labs that may not have access to advanced imaging equipment. By offering a fast, reliable, and objective way to analyze parasite forms, our work supports better disease monitoring and research. It also opens the door for similar approaches to be used in studying other microscopic organisms.

## Introduction

Trypanosomiasis, a disease caused by the protozoan parasite *Trypanosoma brucei* (*T. brucei*), poses a significant global health burden, particularly in sub-Saharan Africa. Human African Trypanosomiasis (HAT) or sleeping sickness, caused by *T. brucei gambiense* and *T. b. rhodesiense* [1], is a neglected tropical disease transmitted by the tsetse fly (*Glossina* spp.) vector [2]. In the mammalian bloodstream, *T. brucei* exhibits pleomorphism, existing in two distinct morphological forms – slender and stumpy [2,3]. The slender forms proliferate rapidly, periodically undergoing antigenic variation to evade the host’s immune response. As the parasitemia progresses, slender forms differentiate into non-proliferative stumpy forms, pre-adapted for transmission to the tsetse fly vector [2]. This density-dependent transition is crucial for regulating parasitemia, prolonging host survival, and facilitating disease transmission [4–6].

The stumpy form exhibits several characteristics that enable its survival in the tsetse fly midgut and subsequent differentiation into the next life cycle stage. These include increased resistance to acidic and proteolytic stress conditions encountered in the fly midgut [7,8]. Additionally, stumpy forms undergo mitochondrial activation and repositioning of cellular organelles, preparing them for the metabolic shift from glucose to proline as the primary energy source in the tsetse fly [7].

Accurate identification and classification of these distinct life cycle stages are essential for understanding *T. brucei’s* biology, transmission dynamics, and developing effective control strategies against trypanosomiasis. Distinguishing slender and stumpy forms is crucial for understanding *T. brucei* infection dynamics, as these stages play distinct roles in disease progression and transmission. Slender forms are proliferative and dominate early infections, while stumpy forms are pre-adapted for uptake by tsetse flies, ensuring parasite transmission. This is why the accurate classification of these forms is highly important for many research labs. Automated classification could further enhance surveillance efforts by providing insights into disease progression and treatment efficacy *in vitro.*

Conventional methods, such as microscopic examination, are time-consuming, labor-intensive, and prone to inter-observer variability [1]. This is why several labs use transgenic markers like PAD1 or PAD2 (https://pmc.ncbi.nlm.nih.gov/articles/PMC2685892/) as stumpy markers, requiring fluorescence microscopy or flow cytometry. However, some of these markers have recently been described as being expressed in intermediate forms rather than being exclusive to fully differentiated stumpy forms (https://pubmed.ncbi.nlm.nih.gov/37824530/). These techniques, while useful, require specialized equipment, trained personnel, and can be challenging to apply in large-scale studies or resource-limited settings.

Recent advances in artificial intelligence (AI) and deep learning (DL) techniques, particularly convolutional neural networks (CNNs), have revolutionized biomedical image analysis tasks across various applications such as disease detection, cellular imaging, and image reconstruction [9–15]. Deep learning approaches have demonstrated expert-level accuracy in medical image analysis, including histopathology and parasite detection, with some models matching or surpassing human performance in microscopic imaging tasks [16].

Previous studies have explored machine learning approaches for the detection of trypanosomes in microscopic images. For instance, de Lima et al. [17] developed a machine learning-based approach for detecting *Trypanosoma cruzi* in blood smears using mobile phone images, while Vandenberghe et al. [18] applied deep learning techniques for the microscopic examination of protozoan parasites. Furthermore, the *Tryp* dataset [19] provides a collection of unstained blood smear images for trypanosome detection. However, these studies primarily focus on trypanosome presence detection rather than classification of distinct life cycle stages. To the best of our knowledge, no prior work has applied deep learning specifically to classify *T. brucei* bloodstream forms into slender and stumpy stages.

In this study, we aim to address this gap by presenting a novel deep learning-based approach for the classification of *T. brucei* life cycle stages using microscopic images. By leveraging the power of CNNs models, we strive to develop a robust system that can accurately identify and distinguish these two morphological stages using *in vitro* parasites. The ability to accurately classify and differentiate the slender and stumpy forms is crucial for several experiment used in the field of *T. brucei*. Our deep learning-based approach provides a rapid, semi-automated, and accurate method for identifying these distinct life cycle stages in microscopic images.

## Materials and methods

### *T. brucei* cell line

**Fig 1:**
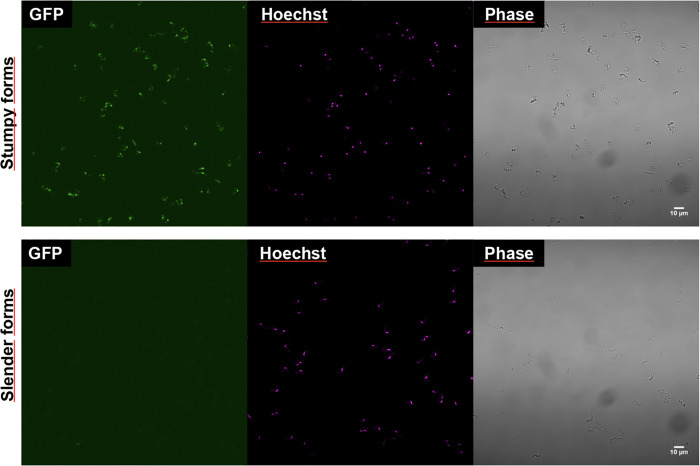
Representative images of slender and stumpy forms captured using widefield fluorescence microscopy, displaying, in order: GFP signal, DNA staining, and phase contrast.

The pleomorphic *T. brucei* EATRO1125 strain, AnTat1.1 clone, expressing a GFP::PAD1–3’UTR reporter, was used in this study. This cell line expresses green fluorescent protein (GFP) under the control of the PAD1 3’-untranslated region (3’UTR), allowing quantification of parasites that have initiated their transition to stumpy forms or that are stumpy forms. Slender bloodstream forms were maintained at a cell density below 5 × 10⁵ cells/mL in HMI-11 medium [20] at 37°C and 5% CO₂. To induce transition to stumpy forms, cultures were allowed to reach high densities of approximately 5 × 10⁶ cells/mL. Stumpy form populations were harvested after 48 hours of high-density culture, exhibiting 99% GFP expression, assessed by widefield fluorescence microscopy, while slender forms exhibited less than 1% GFP expression (Fig 1). We followed the parasite concentrations and timing used by Serra et al. [21] to make sure we got a well and healthy characterized stumpy population.

### Sample preparation for microscopy imaging

**Fig 2:**
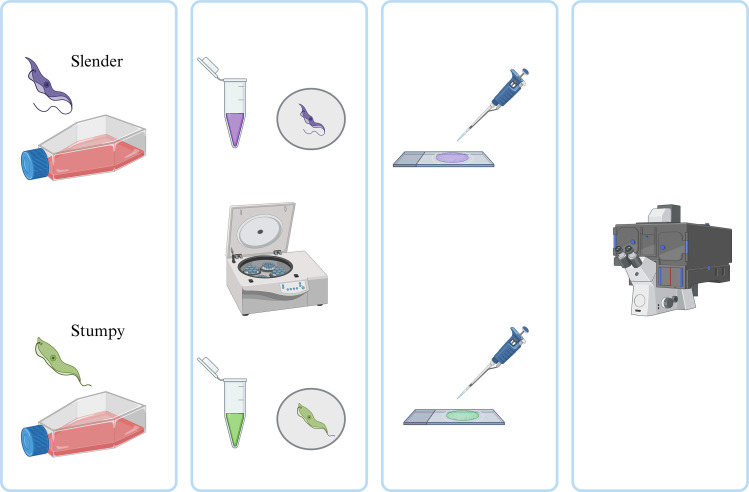
Schematic illustration of slide preparation for microscope imaging. *T. brucei* cultures are grown and maintained in HMI-11 medium (Step 1). Upon reaching the desired cell density, the cultures are centrifuged to concentrate the cells (Step 2). The concentrated cell suspension is then deposited onto glass slides, ensuring optimal cell density without overlapping parasites (Step 3). Finally, the prepared slides are imaged using a Widefield_Fluorescence microscope (Step 4).

Parasite cultures were centrifuged at 1800 rpm for 10 minutes to collect the cells. After removing the supernatant, 250 μL of the concentrated cell suspension was deposited onto glass slides [22], with Merk circular cover glass 12 mm and examined under a microscope to ensure an optimal cell density without overlapping parasites. The slides were then air-dried at room temperature (between 12–18 hours, depending of the total volume). For microscopic imaging, the dried slides were rehydrated in a tap water bath (10 minutes), covered with mounting oil and a coverslip, and sealed with nail polish to prevent dehydration. Fig 2 shows the process of sample preparation for this project. This image was created using BioRender [23].

### Ethics statement

This study does not involve human participants, clinical samples, or patient data. All experimental procedures were conducted using *Trypanosoma brucei* cell cultures, which do not require ethical approval under current institutional and international guidelines.

### Microscopy image acquisition

Images were acquired on a ZEISS Cell Observer widefield fluorescence microscope (Carl Zeiss Microscopy GmbH, Germany) equipped with a Photometrics Prime BSI Express sCMOS camera (Teledyne Vision Solutions, U.S.) using a Plan-Apochromat 63x/1.4 oil immersion objective (pixel size = 0.103 µm). Brightfield images were acquired with transmitted light LED intensity set to 30% and 40 ms exposure time. GFP images were acquired with 475 nm LED excitation, 495 nm dichroic, and a bandpass 500–550 nm emission filter. Hoechst images were acquired with 385 nm LED excitation, 395 nm dichroic, and a bandpass 420–470 nm emission filter. Automatic acquisition in multiple random positions with software autofocusing was set up in ZEN for each coverslip.

### Automated preprocessing pipeline for cell segmentation and post-processing

We developed an automated preprocessing pipeline for cell segmentation and post-processing, which prepares images for subsequent classification.

### Cell segmentation

The first step of the pipeline is cell segmentation. Our method relies on the Cellpose algorithm [24], a widely used versatile tool for accurate segmentation of cells. The algorithm’s pretrained model was employed to accurately identify and segment individual cells from the images. This approach required no additional model training due to the high accuracy and adaptability of the pretrained network, making it well-suited for our diverse set of microscopy images.

### Post-processing

Following cell segmentation, each identified cell was stored as an individual image with dimensions of 299 × 299 pixels. To minimize potential inaccuracies in subsequent analysis steps, cells that overlapped with the original camera image edges by 5 or more pixels were excluded from the dataset. This threshold was selected to ensure that partially visible cells, which might lack complete morphological features, were not included in the classification pipeline. Small particles erroneously detected as cells were also removed to improve data quality. For this purpose, we leveraged Cellpose 3.0 and trained a custom model on a curated subset of 11 randomly selected images containing both true cells and contaminants. The model was trained to distinguish authentic *T. brucei* cells from debris, ensuring that only high-confidence cells were used for classification.

Slender and stumpy forms of *T. brucei* exhibit distinct morphological differences that were considered in the classification process. Slender forms are elongated, skinnier and with a long flagellum. In contrast, stumpy forms are shorter and broader, with a more compact cell body. These morphological differences influence the parasite’s biological function and are critical for accurate classification. During dataset preparation, these features were visually confirmed and used as a basis for annotation to ensure that each cell was correctly labeled before training the classification model. The segmentation and post-processing steps preserved these morphological traits by filtering out incomplete or ambiguous cells, thereby maintaining high-quality input data for deep learning-based classification.

**Fig 3:**
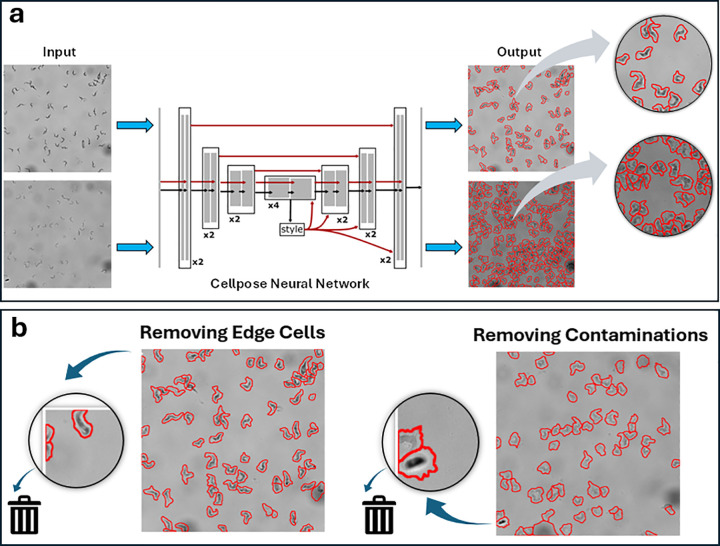
Automated pipeline employed for preprocessing in *T. brucei* life cycle stage classification. The pipeline consists of two main stages: (a) Cell detection and segmentation using the Cellpose algorithm; and (b) Post-processing, which includes the automated removal of cells at the original camera image edges and filtering of potential contaminants mistakenly identified as cells. The raw input images are processed by Cellpose, which then outputs segmented images. This panel is intended to illustrate how the algorithm identifies and delineates individual parasites from the background. The processed cell images from this pipeline are subsequently used for classification.

Fig 3A and 3B demonstrates the automated preprocessing pipeline developed for the first phase of the *T. brucei* classification project. The second phase in the semi-automated pipeline is the classification of *T. brucei* parasites.

### Classification model

We evaluated several deep convolutional neural network (DCNN) architectures, including ResNet50 [25], VGG16 [26], InceptionV3 [27] and Xception [28], for the task of classifying *T. brucei* life cycle stages from microscopic images. The performance metrics for these architectures are summarized in Table 1, highlighting the superior accuracy and computational efficiency of the Xception model.

**Table 1:** Accuracy of Tested DCNN Models for *T. brucei* classification.

DCNN Models	Accuracy
InceptionV3	78%
ResNet50 Model	68%
VGG16 Model	59%
**Proposed Custom Xception Model**	**97%**

The Xception model, based on depthwise separable convolutions, demonstrated the best performance on our dataset and was chosen for the final classification task. This architecture reduces the number of parameters and computational complexity compared to traditional convolutional layers while maintaining high accuracy, making it efficient for our purposes. Furthermore, the observed performance differences reflect the superior feature extraction capabilities of Xception, which we attribute to its ability to capture both global and fine-grained image features.

To further enhance performance, we leveraged transfer learning by initializing the Xception model with pre-trained weights from the ImageNet dataset [29]. Transfer learning allows us to use a model that has already learned rich feature representations from a large-scale, diverse dataset. In this way, the Xception model starts with the ability to identify various low- and mid-level image features, providing a strong foundation that can be fine-tuned for our specific classification task with a smaller, more specialized dataset.

### Custom xception model with transfer learning

To tailor the Xception architecture for the classification of *T. brucei* life cycle stages, we introduced a custom design termed Custom Xception. The standard Xception model, while powerful for general image classification tasks, was not specifically optimized for the fine-grained morphological distinctions required for this study. Therefore, we modified the architecture to enhance its feature extraction capability for our dataset, which presents unique challenges due to the subtle differences between slender and stumpy forms.

Initially, the model was integrated into our custom architecture with all its layers frozen to preserve the general features acquired from ImageNet. This step ensures that the foundational features learned by the model remain intact. However, during the fine-tuning phase, all layers were unfrozen, allowing the model to adapt the pre-learned features more effectively to the intricate and small-scale morphological differences in our dataset. The addition of new layers was guided by our hypothesis that deeper feature representations would improve classification performance. These new layers, comprising a global average pooling layer and a series of fully connected layers, were designed to enhance the model’s ability to capture the complex and specific features of *T. brucei* life cycle stages. Regularization techniques, including batch normalization and dropout, were incorporated to mitigate overfitting. The final layer outputs predictions corresponding to the two life cycle stages of *T. brucei.*

Compared to the original Xception model, our modifications included reducing the depth of the model to optimize for our dataset size and incorporating additional fully connected layers tailored to fine-grained classification. Unlike the standard Xception, which is designed for large-scale datasets with diverse classes, our custom version focuses on distinguishing between two visually similar categories, requiring adjustments in depth, feature extraction emphasis, and regularization strategies.

The performance differences reported in Table 1 further underscore the enhanced feature extraction capabilities of the modified Xception architecture compared to standard architectures such as ResNet-50 and InceptionV3, which have a comparable number of parameters. Our initial exploratory experiments demonstrated that these alternative models either underperformed in capturing the subtle morphological features of *T. brucei* or exhibited higher variance in predictions, justifying the choice of Custom Xception. Additionally, similar modifications in previous studies have led to improved outcomes for fine-grained classification tasks, further supporting our approach.

**Fig 4:**
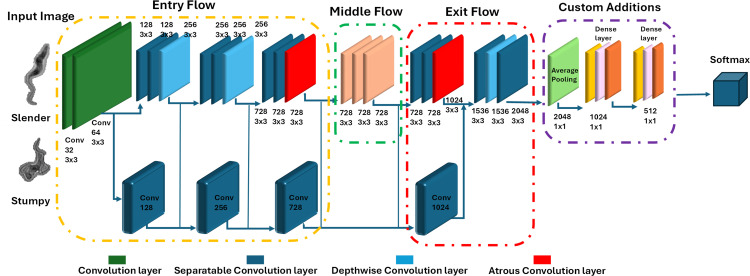
Architecture of the custom xception model for classifying *T. brucei* life cycle stages. The model integrates a pre-trained Xception backbone with additional layers tailored for our classification task. Following the Xception feature extractor, a Global Average Pooling Layer reduces the feature map dimensions. This is followed by one or more Fully Connected Dense Layers, with Batch Normalization applied before the activation functions and Dropout used for regularization. The final Output Layer uses a Softmax Activation Function to classify the parasite cells into slender and stumpy forms. The architecture leverages transfer learning from the ImageNet pre-trained weights, ensuring a robust foundation for accurate classification from microscopic images.

To train the model, we used the Adam optimizer with a learning rate of 0.001 and L2 regularization (weight decay) of 1e-4, which helps prevent overfitting. The model was trained for 30 epochs, where an epoch refers to one complete pass through the entire training dataset. During each epoch, the model alternates between training and evaluation modes, with training and validation losses and accuracies monitored to assess performance. The proposed network architecture, combining the pre-trained Xception model with our custom fully connected layers and fine-tuning strategy, is illustrated in Fig 4.

### Dataset and experimental settings

For this study, we utilized a dataset of microscopic images capturing *T. brucei* cells in their slender and stumpy forms. The original dataset was acquired using a widefield fluorescence microscope, comprised a total of 414 images for the slender class and 414 images for the stumpy class. Using Cellpose 3.0, we counted the number of cells from each class, obtaining a total of 14,325 cells for the slender class and 18,970 cells for the stumpy class.

### Data augmentation

To enhance the training process and mitigate overfitting, we applied several data augmentation techniques specifically designed to increase the diversity and robustness of the training dataset. Each training image had dimensions of 299 x 299 pixels and then was subjected to random horizontal and vertical flips with a 50% probability for each. These common augmentation techniques are frequently used to improve model generalization by simulating variations in image orientation [30–32]. After augmentation, the images were converted to PyTorch [33] tensors and normalized using the standard mean and standard deviation values derived from the ImageNet dataset, ensuring consistency with the pre-trained Xception model’s expectations. For the validation and test datasets, only resizing and normalization were applied, as data augmentation is typically reserved for the training phase to maintain the integrity of validation and test results as data augmentation is generally reserved for the training phase to avoid biasing evaluation metrics.

### Dataset composition and splits

After applying our automated preprocessing pipeline, which involved cell segmentation and filtering steps, we extracted a total of 14,325 slender cells and 18,970 stumpy cells from the original dataset. Data augmentation techniques were applied to significantly increase the size of the training dataset, ensuring the model had sufficient data to learn meaningful features.

To create the training, validation, and test sets, we used a deterministic split based on the ordering of cells in the dataset. Specifically, for the slender class, the first 8,000 cells were allocated for training, the next 3,162 cells for validation, and the subsequent 3,163 cells for testing. Similarly, for the stumpy class, the first 12,570 cells were used for training, followed by 3,200 cells for validation and 3,200 cells for testing. This systematic splitting approach ensures that there is no overlap between the subsets and provides a consistent methodology for reproducibility.

We opted not to use k-fold cross-validation in this study for several reasons. First, the dataset is sufficiently large, and the training, validation, and test splits are representative and non-overlapping, providing a reliable evaluation of the model’s performance. Second, the deterministic split ensures consistency and reproducibility, which is crucial for benchmarking. Third, the performance metrics reported on the held-out test set, such as accuracy and F1-score, offer a robust measure of the model’s effectiveness. While k-fold cross-validation can provide additional insights, its computational demands are substantial for deep learning models. Future work could incorporate k-fold cross-validation to further strengthen the analysis if necessary.

### Experimental settings

The model was trained using the ADAM optimizer, which is a widely used optimization algorithm for deep learning models, known for its computational efficiency and ability to handle sparse gradients and non-stationary objectives. The categorical cross-entropy loss function was employed to optimize the model’s performance during training. Additionally, a learning rate scheduler was implemented to dynamically adjust the learning rate, ensuring efficient convergence and preventing the model from getting stuck in local minima. The batch size was set to 32, and the whole model was implemented on the PyTorch environment using an NVIDIA RTX 3090 GPU platform. The best-performing model was selected after multiple experiments.

#### Loss Function.

The categorical cross-entropy loss function is a widely used loss function for multi-class classification problems in deep learning. It measures the performance of a classification model whose output is a probability distribution over the classes. Let’s define the variables first: N is the number of samples, C is the number of classes, y is the true label represented as a one-hot encoded vector of length C, and p is the predicted probability distribution over the classes, also a vector of length C. The categorical cross-entropy loss function can be formalized as:


$$L=\frac{-1}{N}\sum_{i=1}^{N}\sum_{j=1}^{C}y_{ij}log\left(p_{ij}\right)$$

Where $y_{ij}$ is the true label for sample i and class j (0 or 1 since it’s one-hot encoded), and $p_{ij}$ is the predicted probability for sample i and class j. The outer summation is over the samples, and the inner summation is over the classes. This formulation calculates the negative log probability of the predicted probability $p_{ij}$ for the true class j (where $y_{ij}$ is 1) for each sample i. These negative log probabilities are then summed over all classes and samples, and the average is taken by dividing by the total number of samples N. The goal during training is to minimize this loss function, which encourages the model to assign higher probabilities to the correct classes and lower probabilities to the incorrect classes.

## Results and discussion

### Model performance evaluation

We evaluated the performance of several deep convolutional neural network architectures, including InceptionV3, ResNet50, and VGG16, and our proposed Custom Xception model, for the task of classifying *T. brucei* slender and stumpy forms from microscopic images. Our Custom Xception model, which involved removing the last few layers of the pre-trained network and replacing them with randomly initialized higher-dimensional layers, demonstrated superior performance.

The performance of each model was measured on a held-out test set that was not used during the training or validation processes. The test set comprised 3,163 slender cells and 3,200 stumpy cells, extracted from the original dataset and processed through our semi-automated pipeline. This test set allowed us to evaluate the generalization capabilities of the model on unseen data.

Our Custom Xception model achieved an overall classification accuracy of 97% on this test set. In comparison, the InceptionV3 model attained an accuracy of 78%, while the ResNet50 and VGG16 models achieved lower accuracies of 68% and 59%, respectively.

To comprehensively evaluate the performance of our Custom Xception model, we calculated several key metrics, including accuracy, F1−score, recall, and precision. These metrics were derived from the model’s predictions on the held-out test set, using the following formulas:

Accuracy is the ratio of correctly classified samples to the total number of samples, calculated as:


$$Accuracy=\frac{\left(TP+TN\right)}{\left(TP+TN+FP+FN\right)}$$

Where TP (True Positives) represents the number of correctly classified positive samples, TN (True Negatives) represents the number of correctly classified negative samples, FP (False Positives) represents the number of negative samples incorrectly classified as positive, and FN (False Negatives) represents the number of positive samples incorrectly classified as negative.

The F1−score is the harmonic mean of precision and recall, providing a balanced measure of a model’s performance. It is calculated as:


$$F1-score=\frac{2*(Precesion*Recall)}{\left(Precision+Recall\right)}$$

Precision, also known as positive predictive value, is the ratio of correctly classified positive samples to the total number of samples classified as positive, given by:


$$Precision=\frac{TP}{\left(TP+FP\right)}$$

Recall, also known as sensitivity or true positive rate, is the ratio of correctly classified positive samples to the total number of actual positive samples, calculated as:


$$Recall=\frac{TP}{\left(TP+FN\right)}$$

In the analysis of both the “Slender” and “Stumpy” classes, we observed the following results: For the “Slender” class, there were a total of 3043 true positives (TP), indicating correct predictions, alongside 149 false negatives (FN), which were misclassified as “stumpy.” Additionally, there were 3171 true negatives (TN) reflecting accurate predictions of “stumpy” and 29 false positives (FP), where stumpy instances were incorrectly predicted as “slender.” This led to a precision of approximately 99.06% (Precision = 0.9906) for the slender class, highlighting high accuracy in positive predictions, and a recall of about 95.33% (Recall = 0.9533), which indicates the model’s ability to identify actual slender instances. The F1 score, combining precision and recall, was around 97.16% (F1 = 0.9716), demonstrating strong overall performance despite a higher misclassification rate. In contrast, the “Stumpy” class analysis revealed 3171 true positives (TP) and a low false negative count of 29, suggesting effective prediction of stumpy instances. The true negatives (TN) were reported as 3043, indicating accurate identification of slender instances, while false positives (FP) amounted to 149. The precision for the stumpy class was approximately 95.51% (Precision = 0.9551), showcasing solid predictive accuracy, with a notably high recall of around 99.09% (Recall = 0.9909), reflecting the model’s effectiveness in identifying actual stumpy instances. The F1 score for this class was approximately 97.26% (F1 = 0.9726), indicating exceptional performance and a good balance between precision and recall.

### Confusion matrix and classification accuracy

**Fig 5:**
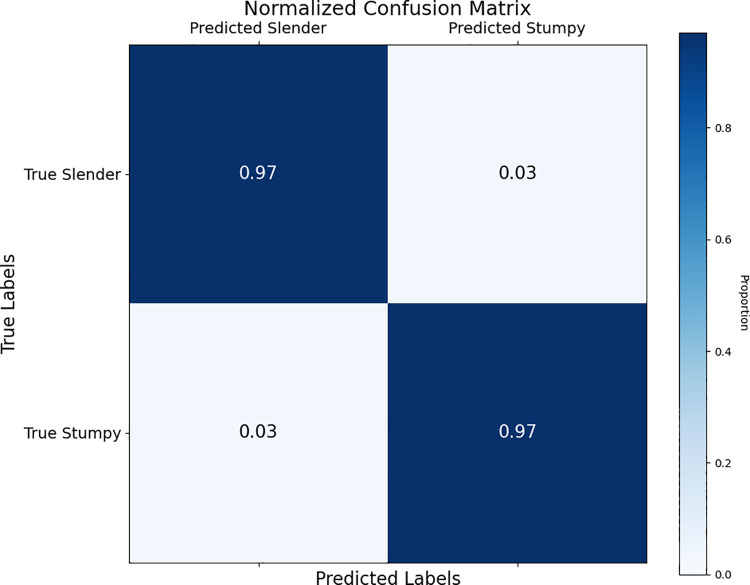
Normalized confusion matrix for the Custom Xception model on the test set. The matrix shows the proportion or percentage of correctly and incorrectly classified samples in each class (slender and stumpy). The model correctly classified 97% of the slender samples and 97% of the stumpy samples, while misclassifying 3% of the slender samples as stumpy and 3% of the stumpy samples as slender.

The normalized confusion matrix (Fig 5) provides a comprehensive understanding of the model’s performance by showing the proportion of correctly and incorrectly classified samples in each category. Our model correctly classified 97% of the slender samples and 97% of the stumpy samples, while misclassifying 3% of the slender samples as stumpy and 3% of the stumpy samples as slender. This level of accuracy indicates that the model is highly effective in distinguishing between slender and stumpy cells, despite the potential challenges posed by imbalanced datasets.

Fig 5 shows the overall proportion of correctly and incorrectly classified samples for both slender and stumpy parasites. We next calculated the confidence levels of the model’s predictions for individual samples.

### Code availability

The code used in this study is publicly available at https://doi.org/10.5281/zenodo.15072493. The provided code is well-documented and commented to facilitate execution, adaptation, and potential modifications by users.

### Classification failure analysis

Despite the overall promising performance of our proposed network architecture, we encountered certain cases where the model failed to classify the *T. brucei* life cycle stages correctly. One contributing factor was the quality of some images: despite the software autofocusing used in image acquisition, some of the images were acquired out-of-focus and appeared blurred. Additionally, in certain instances, multiple parasites were attached to each other, forming a mass. When parts of these conjoined parasites were detected and segmented by the Cellpose algorithm, they were treated as individual samples, leading to incorrect classifications by the network.

**Fig 6:**
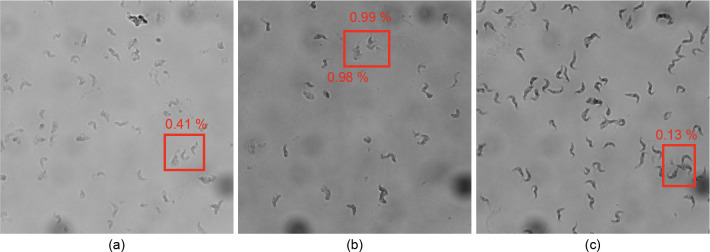
Examples of cases in which the model failed to classify the parasites correctly, leading to misclassification and a drop in performance. Confidence scores are displayed next to the detected parasites. **(a)** Low-quality images with poor contrast and resolution, resulting in saved samples lacking the required features for extraction, causing misclassification. **(b)** Two so-called “dead cells” are misclassified as one of the target classes due to their similar features to *T. brucei* cells. **(c)** Multiple parasites are attached to each other, forming a mass; when a parasite is detected from this mass, it lacks the proper features for extraction, leading to misclassification.

Furthermore, we observed the presence of unknown cells, which we refer to as “dead cells,” that shared some characteristics with *T. brucei* cells. When these dead cells were included in the training or testing samples, the network often misclassified them, as their features deviated from the expected patterns of the target life cycle stages. Fig 6 illustrates examples of these failure cases, including low-quality images, conjoined parasites, and dead cells, which contributed to the misclassifications by our model. While overlapping parasites were rare in the test dataset, we acknowledge that segmentation artifacts, such as incorrectly separating conjoined parasites or failing to fully isolate individual cells, could contribute to classification errors in certain cases.

The observed failure cases highlight potential areas for improvement, such as incorporating additional quality control filters at the segmentation stage to discard poorly focused or ambiguous samples before classification. Future iterations of our pipeline could integrate confidence scores or uncertainty quantification methods to flag low-quality segmentations automatically, enhancing the robustness and reliability of the overall system.

### Sample morphologies and prediction confidence for slender and stumpy cells

**Fig 7:**
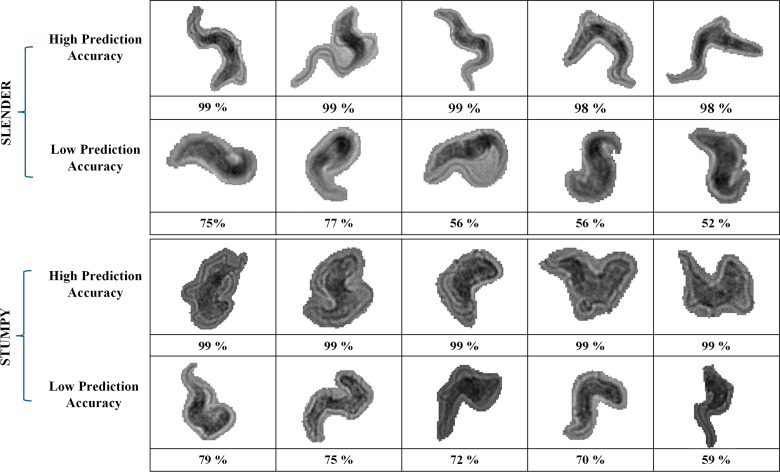
Examples of microscopy images of slender and stumpy forms classified by the Custom Xception model. The top two rows display slender forms, with the first row showing cells classified with the highest accuracy and the second row showing correctly classified slender cells with lower confidence. The bottom two rows represent stumpy forms, where the third row includes high-confidence classifications and the fourth row features lower-confidence, correctly classified stumpy cells. Differences in morphological features contribute to the varying prediction confidence levels of the model.

Fig 7 presents examples of *Trypanosoma brucei* cells from both the slender and stumpy classes, demonstrating how morphological variations affect the prediction accuracy of our Custom Xception model.

The top two rows display slender cells. The first row shows samples with the highest prediction accuracy, as these cells exhibit distinct morphological characteristics of the slender form, making them easier for the model to classify. The second row includes slender cells that, while correctly classified, were done so with lower confidence due to their ambiguous features or partial resemblance to stumpy forms.

The bottom two rows highlight examples from the stumpy form. The third row displays stumpy cells classified with high confidence, clearly representing the morphological traits of the stumpy form. The final row shows stumpy cells classified with lower confidence, likely due to their features being less distinct or resembling slender forms. This figure emphasizes how cell morphology impacts the model’s classification accuracy, with clearer, more distinctive features leading to higher prediction confidence, while ambiguous features result in lower confidence.

### On image resolution and robustness

We acknowledge the concern regarding the resolution of the images. It is important to note that not every laboratory has access to high-end, expensive microscopes capable of producing high-resolution images. One of the goals of our study was to develop a robust classification model capable of accurately distinguishing *T. brucei* life cycle stages, even when working with lower-resolution images.

The ability of our Custom Xception model to classify cells accurately despite the low resolution of the input images highlights its robustness and practical applicability. This feature is particularly advantageous for resource-limited settings, where access to high-resolution imaging may not be feasible. By demonstrating strong performance on low-resolution images, our approach broadens the accessibility and potential impact of semi-automated parasite classification methods in diverse laboratory environments.

While low-cost microscopes may provide sufficient resolution for manual visual assessment, distinguishing between slender and stumpy forms remains challenging—even for experienced researchers. Our model enhances classification accuracy by systematically analyzing morphological features that may be subtle or ambiguous to the human eye. By leveraging deep learning, it offers a consistent and objective evaluation of parasite populations, minimizing observer bias and improving reproducibility across laboratories.

## Conclusion

In this study, we developed a fully automated pipeline for the segmentation and classification of *Trypanosoma brucei* mammalian life cycle stages, slender and stumpy forms, from microscopic images. Our approach combined the Cellpose segmentation algorithm with a customized deep learning classification model based on the Xception architecture, enhanced through transfer learning and fine-tuning techniques.

The Cellpose algorithm effectively segmented individual parasites from microscopic images, facilitating the extraction of samples for subsequent classification. For the classification task, we employed a transfer learning strategy by initializing the Xception model with pre-trained weights from the ImageNet dataset. This strategy allowed us to leverage the comprehensive feature representations learned from a large-scale dataset, providing a robust foundation for our specific classification task. Our customized Xception model retained the full architecture of the pre-trained base model while incorporating additional layers tailored to our specific task. We added a global average pooling layer and fully connected layers, which included batch normalization and dropout for regularization. This design allowed the model to adapt effectively to the *T. brucei* dataset while minimizing the risk of overfitting, leading to a robust and accurate classification performance.

Our results demonstrate that deep learning can effectively differentiate between the slender and stumpy bloodstream forms of *T. brucei*, achieving a high classification accuracy of 97% on the test set. However, we acknowledge that the morphological similarities between these two forms pose classification challenges, particularly in ambiguous cases. Future work could explore integrating additional morphological features, such as cell shape descriptors or texture analysis, alongside deep learning-based classification. Furthermore, uncertainty quantification techniques, such as Monte Carlo dropout or ensemble learning, could be implemented to assess model confidence and improve robustness in challenging cases.

Unlike previous studies that primarily focused on detecting *Trypanosoma* parasites in microscopic images, our study focusses more on developing a deep learning-based approach specifically designed for classifying *T. brucei* bloodstream forms for *in vitro* parasites. To the best of our knowledge, this is the first deep learning model tailored for this classification task. By demonstrating the feasibility of automated classification, our work provides a foundation for further developments in parasite stage differentiation, which could be used in research to understand better the biology and characteristics of this parasite. Future research could explore expanding the dataset to include other life cycle stages or *in vivo* samples, thereby increasing the model’s versatility and applicability.

It is important to note that our current study is designed for classifying *T. brucei* bloodstream forms from *in vitro* systems. As such, we do not assume that the model will be used as a standalone screening tool but rather as an assistive method for analyzing lab generated samples. The application of deep learning models to real-world scenarios where the majority of samples are parasite-free presents additional challenges. Since deep learning models may exhibit degraded performance when encountering data distributions that differ from their training set, future work should explore the model’s generalizability to broader screening applications, including its performance on uninfected blood samples. This would be particularly important if the method were to be adapted for large-scale field studies, where the majority of samples may not contain parasites. Additionally, a pre-classification step could be considered to ensure that only parasite-containing images are analyzed by the model, further improving its practical utility.

Overall, our automated pipeline demonstrates the significant potential of deep learning in biomedical image analysis, not only for classifying *T. brucei* life cycle stages but also for broader applications in the classification of other single cells based on their unlabeled morphology. By automating segmentation and classification processes, this approach can enhance large-scale analyses of cellular images, contributing to improved life cycle stage classification in various biological fields. Further enhancements are necessary to address remaining challenges and to achieve even greater accuracy and robustness in wild field applications.

## Supporting information

S1 Fig Supporting materials. (ZIP)
